# Supplementary material for: Complexome profiling of the Chlamydomonas psb28 mutant reveals TEF5 as an early PSII assembly factor
Source: Plant Cell. 2025 Mar 17;37(6):koaf055. doi: 10.1093/plcell/koaf055 (PMC12164590; doi:10.1093/plcell/koaf055)
Supplement: koaf055_Supplementary_Data [file koaf055_supplementary_data.zip › psb28_interactive.zip!/plots/intensity/qualityPlots/peptides/RAB20_Cre11.g482900.t1.1.html]

### Cre11.g482900.t1.1

Trivial name: RAB20  
SeqCoverage [median]: 5.00  
Mass: 21.94 kDa  
  
 

| Sequence | MissedCleavage | Mass [Da] | wtIntensity | muIntensity | Score |
| --- | --- | --- | --- | --- | --- |
| FRTLTSSYYR | 1 | 1292.65 | 351.52 | 0.00 | 68.54 |
| TLTSSYYR | 0 | 989.48 | 10099.04 | 31211.30 | 116.88 |
|  |  |  | 10450.6 | 31211.3 |  |
